# Sarcopenia: recent advances for detection, progression, and metabolic alterations along with therapeutic targets

**DOI:** 10.1139/cjpp-2024-0201

**Published:** 2024-08-26

**Authors:** Syeda Roohina Ali, Augustine T. Nkembo, Srinivas M. Tipparaju, Muhammad Ashraf, Wanling Xuan

**Affiliations:** Department of Pharmaceutical Sciences, USF Health Taneja College of Pharmacy, University of South Florida, Tampa, FL, US

**Keywords:** sarcopenia, metabolism, aging, biomarker, exercise

## Abstract

Sarcopenia, a disorder marked by muscle loss and dysfunction, is a global health concern, particularly in aging populations. Sarcopenia is intricately related to various health conditions, including obesity, dysphagia, and frailty, which underscores the complexity. Despite recent advances in metabolomics and other omics data for early detection and treatment, the precise characterization and diagnosis of sarcopenia remains challenging. In the present review we provide an overview of the complex metabolic mechanisms that underlie sarcopenia, with particular emphasis on protein, lipid, carbohydrate, and bone metabolism. The review highlights the importance of leucine and other amino acids in promoting muscle protein synthesis and clarifies the critical role played by amino acid metabolism in preserving muscular health. In addition, the review provides insights regarding lipid metabolism on sarcopenia, with an emphasis on the effects of inflammation and insulin resistance. The development of sarcopenia is largely influenced by insulin resistance, especially with regard to glucose metabolism. Overall, the review emphasizes the complex relationship between bone and muscle health by highlighting the interaction between sarcopenia and bone metabolism. Furthermore, the review outlines various therapeutic approaches and potential biomarkers for diagnosing sarcopenia. These include pharmacological strategies such as hormone replacement therapy and anabolic steroids as well as lifestyle modifications such as exercise, nutrition, and dietary changes.

## 1. Introduction

### 1.1. Definition and overview of sarcopenia

One of the major global concerns among nations including the USA is the rise in the number of patients suffering from sarcopenia. The cost of treating patients with this condition continues to rise and has led to the hypothesis that preventative therapy would be a better option. The total estimated cost of hospitalizations in individuals with sarcopenia in a study conducted in 2019 was USD $40.4 billion (Goates et al. 2019). Understanding the etiology and progression of sarcopenia are crucial for the improvement of treatment and quality of life in the elderly. Given that sarcopenia is a metabolic disease that interferes with muscle homeostasis, metabolomic studies have the potential to predict the onset of diseases (Miyamoto et al. 2021).

Sarcopenia is a geriatric condition characterized by a progressive loss of muscle mass and function and has been associated with several adverse health condition including fracture, functional decline, and mortality (Cruz-Jentoft and Sayer 2019). In 2010, the European Working Group on Sarcopenia in Older People (EWGSOP) defined sarcopenia using muscle mass, muscle strength, and physical performance (Cruz-Jentoft et al. 2010). In clinical practice, the EWGSOP2 recommends that a person with low muscle strength and low muscle mass or quality should be diagnosed with sarcopenia. The condition can be best understood as skeletal muscle failure or insufficiency (Cruz-Jentoft 2016). Clinicians often associate sarcopenia with leanness and hence overlook the fact that it can also occur in cases of obesity, which can result in heightened muscles disability and mortality. Sarcopenic obesity is usually identified when both low muscle mass and increased adiposity are present in an individual. This condition most often, progress unnoticed when the focus of care is obesity, leading to adverse outcomes (Scott et al. 2014). Sarcopenia and obesity share some underlying pathophysiological pathways (Batsis and Villareal 2018). Muscle loss could increase the risks of disability and even death during weight loss in obese individuals (Cava et al. 2017). However, a universally accepted definition of sarcopenic obesity is still lacking, and the role of muscle strength in diagnosing these patients remains unclear. Furthermore, a connection has been found between sarcopenia and dysphagia (known as sarcopenic dysphagia), which necessitates a distinct approach in clinical practice (Barazzoni et al. 2018).

Sarcopenia diagnosis is relatively straightforward and requires measurement of a combination of muscle mass, muscle strength, and physical performance. Sarcopenia definitions typically require at least two parameters: muscle mass and muscle strength. The different cutoff points for these parameters lead to a lack of standardization. This lack of a standardized definition means that clinicians might diagnose and treat sarcopenia differently depending on which guidelines they follow. This can lead to inconsistent patient care and difficulty in comparing research studies (Bruyère et al. 2016). The updated EWGSOP2 proposed a stepwise approach for diagnosis. Firstly, diagnosis should start with a measurement of muscle strength, usually grip strength, which has a well validated protocol (Roberts et al. 2011). Secondly, the diagnosis should proceed with the measurement of muscle mass. Several techniques have been used to measure muscle mass, yet they all possess significant drawbacks, such as variability in outcomes, inconsistent application of cutoff points, and a tenuous correlation between muscle mass and negative health consequences (Manini and Clark 2012). Due to the variability in the results from the techniques to diagnose sarcopenia, the most efficient and generally acceptable method currently available involves the use of dual-energy X-ray absorptiometry, which provides close-to-correct estimate values of lean mass. Other techniques such as bioelectrical impedance analysis, computed tomography (CT), and MRI are also applicable in certain scenarios to evaluate the indicators of sarcopenia (Buckinx et al. 2018). Most importantly, it is worth noting that MRI and CT scans techniques are widely recognized as the gold standards for measuring muscle mass due to the reliability of their results (Qaisar et al. 2021). Despite CT and MRI being the gold standards for evaluating muscle mass and ensuring accurate assessment of various bodily components, there is disagreement over the cut-off values for sarcopenia.

### 1.2. Metabolic perspectives on sarcopenia

Metabolites are intimately associated with cellular phenotypes, serving as the downstream expression of the transcriptome, proteome, and genome. Mass spectrometry-based metabolomics is a highly reliable method for methodically examining the metabolic profiles of tissues, biological fluids, and cells (Ceglarek et al. 2009). The measurement of metabolic flux is an important parameter for determining the status of metabolic diseases, aging (Gu et al. 2015), and longevity (Montoliu et al. 2014). The knowledge of alterations of metabolites in metabolite profiles is crucial and could enhance comprehension of the pathophysiological processes (Dai et al. 2021). Metabolomics has the potential to be a useful tool in comprehending the metabolic disturbances linked to sarcopenia and can also relate to the degree of the illness (Marques et al. 2023). Sarcopenia has been widely investigated by many people. Previous reports demonstrate the use of predictive biomarkers in sarcopenia, sarcopenic obesity, and metabolism in sarcopenia that contributed to the present understanding in the field. Since sarcopenia is mainly an age-related and geriatric illness, it has been linked to several metabolic conditions, such as obesity, insulin resistance (IR), diabetes, dyslipidemia, and hypertension. In a meta-analysis conducted by Zhang et al. (2018), sarcopenia and metabolomic syndrome were found to be positively correlated, the total prevalence of metabolic syndrome (MS) in middle-aged and older, non-obese persons with sarcopenia was 36.45% (Zhang et al. 2018).

Furthermore, increased levels of the inflammatory cytokines tumor necrosis factor-like weak inducer of apoptosis and tumor necrosis factor alpha (TNF-*α*) are associated with a heightened risk of sarcopenia. Conversely, metabolic hormones such as insulin growth factor 1, insulin, and adiponectin are correlated with a reduced risk of sarcopenia (Li et al. 2019). In a recent study by Marques et al. (2023), it was reported that the plasma metabolic profile showed a correlation with various measurements of sarcopenia. These data suggest a connection between mitochondrial dysfunction due to reduced insulin-like growth factor 1 (IGF-I) levels and sarcopenia, and they illustrate several potential metabolic pathways that could play a role in the etiology of the disease (Marques et al. 2023). Damages induced by reactive oxygen species (ROS) are among the numerous factors that can result in mitochondrial dysfunction or damage. Age-related muscle denervation could be a secondary contributor to the increased ROS production associated with aging, due to the declining levels of antioxidant enzymes (Alway et al. 2017). Previous findings have indicated that with aging, both in human and animal models, there is a decrease in the levels of plasma IGF-I and growth hormones (Jarmusch et al. 2021). Earlier studies and meta-analysis showed that there is a substantial correlation between sarcopenia in individuals over 65 and the metabolic risk factors such as, body mass index (BMI), systolic blood pressure, diastolic blood pressure, triglycerides (TG), fasting glucose, homeostasis model assessment of IR, and high density lipoprotein (HDL), while the risk is larger in males than in women (Carcelen-Fraile et al. 2023).

In this review we discuss the metabolic processes that lead to sarcopenia, with an emphasis on the metabolism of proteins, fats, carbohydrates, and bones. The review provides insights into key questions “how muscle health is impacted by amino acid balance” as well as “how leucine and other amino acids help to increase the synthesis of muscle protein”. We also discuss “how lipid metabolism affects sarcopenia, emphasizing the roles of IR and inflammation”. IR and its role in the development of sarcopenia are examined in connection to glucose metabolism. The paper also discusses how sarcopenia and bone metabolism interact, highlighting the connection between bone and muscle health. Overall, the review covers key components and current understanding with regard to sarcopenia and evaluated the possible biomarkers for the diagnosis of sarcopenia and treatment strategies, including exercise, diet, and the use of pharmacological agents like hormone replacement therapy and anabolic steroids.

## 2. Metabolic dysfunction in sarcopenia

### 2.1. Sarcopenia and protein metabolism

Healthy muscles require a balance in the amino acid and changes in the amino acid profiles therefore may serve as key biomarkers in the diagnosis of sarcopenia. Gaining insight into the variations in the amino acid composition allows determination of pathophysiological pathways (Dai et al. 2021) and previous research offers insights and demonstrated a correlation between decreased muscle mass in elderly persons and alterations in the plasma amino acid composition (Lustgarten et al. 2014). Among the nutrients that humans consume, amino acids are the main nutrient that is directly related to building and maintenance of muscle proteins. Specifically, the essential amino acid, leucine serves as a stimulatory signal and therefore dietarily nutrients that are rich in leucine provide older people to overcome their anabolic resistance and successfully boost the synthesis of muscular protein (Kobayashi 2018).

Glutamic acid and aspartic acid are key amino acids that regulate muscle mass and strength (Zhao et al. 2018), previous evidence suggests that decreased blood levels of branched-chain amino acids (BCAAs), particularly leucine, are linked to reduced strength, and a lower skeletal muscle index (Ter Borg et al. 2019). However, another study identified that participants with low muscle quality had significantly higher levels of isoleucine, leucine, tryptophan, serotonin, and methionine than participants with high muscle quality. This finding may be related to impaired amino acid metabolism, which lowers skeletal muscle uptake and raises plasma amino acid levels in the blood (Moaddel et al. 2016). While leucine has been shown to have numerous beneficial impacts on metabolism across various organs, obesity and IR have been linked to increased amounts of this amino acid, as well as other BCAAs and their metabolites. The underlying cause of the higher plasma BCAA levels in T2D patients and people with IR has not been determined by several research. An underlying cause could conceivably be dysfunctional BCAA catabolism (Moghei et al. 2016; Vanweert et al. 2022). The build-up of several BCAA-catabolic metabolites in the plasma of insulin-resistant individuals with obesity or type 2 diabetes, such as BCAA-derived acylcarnitines (C3 and C5), 3-hydroxyisobutyrate, 2-hydroxbutyric acid, and 2-ketobutyric acid, may be explained by dysfunctional mitochondrial BCAA catabolism (Lerin et al. 2016). More research is necessary to determine whether plasma concentrations of BCAA, particularly leucine, are possible biomarkers of sarcopenia and can provide information beyond dietary evaluations. As of right now, there is no established minimum or ideal plasma concentration of BCAA needed to maintain SM mass (Ottestad et al. 2018).

In a cross-sectional investigation conducted by Dai et al. (2021), the BCAAs leucine, isoleucine, and aromatic amino acid tryptophan demonstrate inverse relationships with sarcopenia. These findings reveal the etiology of sarcopenia and aid in its prevention and therapy. Tryptophan is a metabolite of serotonin and is significant for maintenance of muscle mass and animals lacking tryptophan exhibited considerable muscular atrophy and low growth hormone levels (Lin et al. 1988). According to one study, tryptophan can stimulate the expression of myogenic factors (myogenin, myoD, and myosin heavy chain) in C2C12 myoblasts in vitro and stimulate skeletal muscle IGF-1/p70s6k/mammalian target of rapamycin (mTOR) signaling in vivo. When combined with exercise training, leucine supplements may increase the synthesis of proteins, reduce the breakdown of proteins, and reduce inflammation (Xia et al. 2017). A recent study observed that the plasma metabolic profile varies with different indices of sarcopenia. Citrulline, very long-chain fatty acids (FAs), and carn. Dicarboxylic acid (DC) were identified as significant variables associated with the severity of the condition. These findings suggest a probable link between mitochondrial dysfunction and sarcopenia, highlighting several potential metabolic pathways that could contribute to the disease’s etiology (Marques et al. 2023).

The relationship between different amino acids and sarcopenia, the age-related loss of muscle mass and strength, has been investigated. Studies show that a higher incidence of sarcopenia is linked to lower levels of glutamic acid (Nakajima et al. 2023). Research has shown that glutamic acid is consistently associated with sarcopenia-related traits, such as muscle mass and strength, across different age groups, sexes, and races (Zhao et al. 2022). Regarding aspartic acid, its association with sarcopenia appears more complex. Some studies have found that aspartic acid is linked to sarcopenia primarily in younger individuals (Zhao et al. 2022), suggesting that the relationship might be age-dependent. Aspartic acid was associated with muscle mass and strength in some studies but not consistently across all cohorts (Zheng et al. 2024). Therefore, while glutamic acid seems to have a more consistent link to sarcopenia, aspartic acid’s role is less clear and may vary depending on specific conditions or populations.

Skeletal muscle glutamate also contributes to a number of metabolic processes, including the tricarboxylic acid cycle, purine nucleotide cycle, insulin synthesis, and glutathione synthesis (Rutten et al. 2005). Furthermore, the enzyme glutamic acid decarboxylase can transform glutamic acid into *γ*-aminobutyric acid (GABA), the most prevalent inhibitory transmitter in the brain. It has been observed that taking GABA orally increases the synthesis of muscle proteins and growth hormone, which may help with dynamic amino acids turnover (Sakashita et al. 2019).

The amino acids histidine and beta-alanine combine to form carnosine, a dipeptide that is found in large amounts in the skeletal muscle of mammals. *β*-alanine and histidine can be converted into carnosine by the enzyme carnosine synthase in skeletal muscle cells. It is interesting to note that muscle carnosine loading improves both skilled and untrained individuals’ performance during high-intensity exercise (Derave et al. 2010). Furthermore, carnosine may be able to inhibit a number of the biochemical alterations that come with aging, including diseases linked to protein oxidation, glycation, and cross-linking. Muscle strength and carnosine levels appear to have a complex relationship that may differ between men and women. Higher carnosine levels may be linked to improved muscle strength and function in women, according to certain studies (Zhao et al. 2022). Conversely, the relationship between carnosine levels and muscle strength in men is less clear; some studies suggest that higher carnosine levels may not always translate into stronger muscles (Baguet et al. 2012).

### 2.2. Sarcopenia and lipid metabolism

One of the characteristics of sarcopenia and aging is muscular fat infiltration. Aging, type 2 diabetes, and obesity are all associated with changes in the metabolism of FAs, with lipid buildup inside muscle cells playing a role in the development of ceramides and muscle IR. These lipids consist of polyunsaturated fatty acids (PUFAs), intramyocellular lipids, lipid droplets, and diacylglycerol (Al Saedi et al. 2022). Adipocytes in sarcopenic muscle exhibit decreased activation of PPAR*α-* and ATGL-mediated lipid signaling pathways in comparison with healthy muscle. As a result, proinflammatory cytokines such interleukin-6 and tumor necrosis factor alpha is upregulated, underscoring the critical function of ATGL in reducing age-related inflammation in sarcopenia (Lettieri Barbato et al. 2014).

Although an increase in fat mass is frequently linked to a decrease in muscle mass, sarcopenia is not always connected with weight loss. Skeletal muscle is essential for mobility and plays a critical role in both whole-body energy expenditure and systemic metabolism (Izumiya et al. 2008). Fat serves as a necessary substrate for energy both at rest and during exercise, for muscle contraction. Triglycerides are the primary source of free FAs that are oxidized during exercise, and triglycerides are stored in adipose tissue and inside muscle fibers (Ranallo and Rhodes 1998). It has been suggested that by controlling the growth, proliferation, and differentiation of muscle cells, FAs, and lipid metabolites can control the mass and function of skeletal muscle. Laboratory studies suggest that C2C12 cells exposed to saturated FA palmitate has been observed that leads to a reduction in myotube diameter (Bryner et al. 2012) suggesting that exposure to FAs decreases muscle size.

The links between dyslipidemia and sarcopenia are not well understood; however, some studies in this field suggest that oxidative stress and inflammation play a major role. Clinical studies conducted by others as well as our group reported that CRP levels were greater in sarcopenia patients (Bano et al. 2017) and these studies also provide insights into a plausible connection between low-grade chronic inflammation and sarcopenia. In addition, studies show that increasing body fat causes release of pro-inflammatory cytokines such as TNF-*α* and interleukin-6 which modulates intercellular communication and results in IR, and causes breakdown of proteins in the skeletal muscle (Li et al. 2019).

Sarcopenia and aging both are characterized by the migration of fat into the muscle. The association between obesity and osteoporosis was found to be orchestrated by sarcopenia (Rondanelli et al. 2014). In the elderly lipid metabolism is linked to obesity as measured by BMI and numerous diseases link BMI as a risk factor for heart disease and diabetes (Powell-Wiley et al. 2021). Wang, et al. report that mild cognitive impairment and sarcopenia were substantially linked to lower dietary intake of various phospholipids (PC and SM), unsaturated FAs monounsaturated fatty acid (MUFA), saturated fatty acid (SFA), phosphatidylcohine (PC), and choline types (Wang et al. 2022). Another study found that, when kept within normal reference limits, an increase in lipid metabolism-related parameters (BMI, TG, total cholestrol (TC), and low-density lipoprotein (LDL)) may offer protection against sarcopenia (Jiang et al. 2023). In a cross-sectional study, it was noted that there is an increase in the prevalence of sarcopenia with increasing age, decreased BMI, and fat percentage. Overall, these studies identified the risk variables for sarcopenia, which included higher levels of LDL and homocysteine, male sex, older age, lower levels of TC, TG, and BMI. Therefore, demonstrating that, when the risk factors are kept within normal reference limits, an increase in lipid metabolism-related parameters (BMI, TG, and TC) may offer protection against sarcopenia. Earlier studies shed light on the role and direction of parameters connected to lipid metabolism in preventing sarcopenia (Jiang et al. 2023).

Nonetheless, a number of alternative pathways are proposed to explain this cellular build-up of FAs in muscle, such as the correlation between hormone-sensitive lipase (IMCL) and insulin insensitivity, inflammation, myosteatosis through fibro/adipogenic progenitors, and the build-up of the sphingolipid ceramide, which adversely affects the function of skeletal muscle (Hamrick et al. 2016). Serine/threonine kinases that are activated by FA intermediates hinder the insulin receptor’s capacity to activate downstream substrates like IRS-1. As a result, there is less glucose taken up by skeletal muscle cells since glucose transporter 4 translocation develops IR (Summers et al. 1998). Taken together, the role of fat and lipid metabolism, are closely linked with aging and sarcopenia leading. The cellular pathways and metabolism are linked to decreased muscle size and increased fat accumulation, inflammation, and oxidative stress.

### 2.3. Sarcopenia and glucose metabolism

Skeletal muscle in healthy people responds well to insulin and can absorb up to 85% of circulating glucose when blood glucose levels suddenly rise. Since the availability of glucose and insulin secretion from *β* cells are closely correlated, blood glucose levels can be precisely adjusted to stay within a normal range. Some people gradually lose their capacity to control their blood sugar levels or worsening glucose tolerance as they get older (Broughton and Taylor 1991). Moreover, sarcopenia’s lack of myokines may contribute to development of IR. One of the main deficiencies modulating the relationship between obesity and type 2 diabetes is skeletal muscle (IR) (Cleasby et al. 2016). Sarcopenia increases IR, which in turn causes diabetes and MS. Even in the absence of obesity coexisting, sarcopenia may be an early indicator of IR, diabetes, and multiple sclerosis (Moon 2014). Previous studies from our laboratory demonstrated using diabetic mouse model that IR decreases muscle strength and function, whereas restoring metabolism via targeting nicotinamide phosphoribosyl transferase leads to improved skeletal muscle function in diabetes (Manickam et al. 2022; Wagner et al. 2022).

Additionally, during aging, the capacity of the muscle mitochondria to catalyze the metabolism of FAs decreases, speeding up the development of IR linked to obesity and problems with glucose metabolism (Sivitz and Yorek 2010). Previous studies reported that the individuals with sarcopenia had a strong negative connection between the body weight and appendicular skeletal muscle mass and fasting hyperglycemia (Buchmann et al. 2016). However, the research by Choi et al. revealed that among the female group, fasting blood glucose levels were within normal ranges for women with sarcopenic obesity (Choi and Park 2016). It has been shown that with sufficient intakes of leucine and protein, along with load bearing and endurance exercise, muscle mass and function can be restored and maintained close to youth levels. Both signals from the gut microbiota and optimal muscle function enhance the muscle’s ability to dispose of glucose, which helps with glucose management. Additionally, consuming non-digestible carbohydrates produces short-chain FAs, which enhance glucose control and promote muscle building (Daily and Park 2022). A higher likelihood of sarcopenia presentation is associated with MS or its risk factors including hypertriglyceridemia, increased waist circumference, low HDL cholesterol, arterial hypertension, and fasting blood glucose. with higher risk in men compared with women (Kassi et al. 2011).

The functions of known putative molecular mediators in the metabolism of protein and glucose in skeletal muscle and the reported effects on signaling pathways and effector machinery (glucose transporters, mitochondrial function, translation, and activation of E3 ubiquitin ligases), related to glucose and protein metabolism include those of insulin, insulin-like growth factor 1 (IGF1), amino acids, myostatin, urocortins, and vitamin D (Cleasby et al. 2016).

### 2.4. Sarcopenia and bone metabolism

Given the likelihood that older adults with osteopenia/osteoporosis also have sarcopenia, and vice versa, low bone mass is clinically diagnosed as osteopenia/osteoporosis (Miyamoto et al. 2021). Notably, there is evidence that those over 50 who are diagnosed with obesity and sarcopenia at the same time are more likely to develop osteoporosis than those who are diagnosed with obesity or sarcopenia alone (Waters et al. 2010). This critical association holds true even after accounting for sex, age, and degree of activity. Research shows that adding adipose tissue to a high-muscle phenotype does not increase body mass or improve bone mineral density (Sowers et al. 1992). This is not surprising, as it is well known that strain magnitude plays a major role in determining bone density and strength (Rubin and Lanyon 1985), and that strain magnitude is significantly decreased with loss of muscle mass and strength (Hunter et al. 2019).

Diagnostic techniques that could distinguish between sarcopenia and bone metabolism and accurately predict fractures and falls would be very effective in clinical practice. CT was tested as a useful tool for measuring intermuscular fat and muscle quality to determine the amount and quality of skeletal muscle (Kuriyama et al. 2022). These disorders increase the risk of many negative outcomes, which could be avoided if both are detected and treated. This is especially true for older persons, for whom the diagnosis of one should prompt the search for the other (Duque et al. 2023). Several studies stated that in older adults, taking creatine supplements and doing resistance exercise improves lean mass and muscle strength while lowering bone catabolism markers without having a negative impact on liver or kidney function. Nevertheless, if exercise intervention is not performed, these advantages vanish. In one of the studies, it was discovered that elevated glycine and reduced taurine levels were indicative of the onset of osteoporosis and sarcopenia (Miyamoto et al. 2021).

The skeletal muscle secretome has become a crucial hub for the detailed investigation of the metabolic relationship between aging muscles and bone health, and it may offer fresh perspectives and viable therapeutic approaches for the management of metabolic disorders (Liu et al. 2022). To release a range of cytokines that facilitate communication between muscle and bone, muscle, and bone function as endocrine organs. Myokines secreted by muscles, including IL-6, irisin, BAIBA (*β*-aminoisobutyric acid), and myostatin, regulate osteoblast and osteoclast processes and impact bone metabolism. Bone secretes osteoprotegerin, wnt-3a, sclerostin, fibroblast growth factor 23 (FGF-23), and other osteokines that govern muscle metabolism. A thorough understanding of how myokines interact with muscles and bones can be used to create individualized prevention or treatment programs for illnesses associated with them (Shao et al. 2024). Skeletal muscle and myokines involved in bone absorption and resorption can be impacted by exercise, aging, and inactivity. It seems that exercise with a sufficient mechanical stress is a powerful way to increase bone mineral density (BMD), strength, and bone mass (Gries et al. 2022). Muscle and bone interact, and muscle produces myokines that control bone homeostasis (Schnyder and Handschin 2015). For instance, exercise-induced muscle myokine irisin regulates bone metabolism (Kim et al. 2018). It has been discovered that muscles generate and secrete extracellular matrix (ECM) vesicles that control bone homeostasis (Kim et al. 2018). Bone-remodeling factor (IGF1), contained in ECM proteins in bones and produced by osteoblasts, is released during osteoclastic bone resorption and stimulates subsequent osteoblastic bone-forming activities (Xian et al. 2012). In muscles, IGF1 also promotes anabolic and inhibits catabolic pathways (Miyamoto et al. 2021). Maintaining skeletal homeostasis and bone mass during bone remodeling is one of IGF-1’s fundamental roles in the bone matrix (Xian et al. 2012). Another myokine, irisin tends to promote osteoblast activity, which in turn activates activating transcription factor 4 (ATF4), which appears to have a beneficial influence on bone development (Colaianni et al. 2016). Recently identified as a myokine with numerous beneficial properties, BAIBA, together with GABA, is shown to be positively related to BMD and inversely related to osteoporosis risk (Wang et al. 2020). Given the overall positive effect of exercise on bone mass, it is likely that these pro-inflammatory myokines from exercise have less of an effect than the anti-inflammatory myokines on bone in the context of skeletal muscle to bone communication during and after exercise (Gries et al. 2022).

### 2.5. Impact of age and gender differences on metabolic dysregulation and their correlation to sarcopenia

Age and gender differences play a significant role in metabolic dysregulation and their correlation to sarcopenia. With aging, both men and women experience changes in muscle mass and strength, but the patterns and impacts of these changes can differ (Palmer and Jensen 2022). Men typically have higher muscle mass and strength compared to women, but they also experience a more rapid decline in muscle mass with age. This accelerated loss can lead to increased risk of sarcopenia in older men. Women, on the other hand, generally have lower muscle mass and strength throughout life, but their rate of muscle loss tends to be more gradual (Bartolomei et al. 2021). Gender differences in hormone levels, particularly testosterone in men and estrogen in women, significantly affect muscle mass and metabolic function. Testosterone promotes muscle growth and maintenance in men, while estrogen has protective effects on muscle mass in women. As men age, the decline in testosterone levels contributes to the loss of muscle mass and strength. Similarly, postmenopausal women experience a decrease in estrogen levels, which can lead to an increased risk of sarcopenia (Palmer and Jensen 2022). Metabolic dysregulation, such as IR and chronic inflammation, is commonly associated with sarcopenia. These conditions are influenced by both age and gender. Older adults often experience IR, which impairs glucose metabolism and contributes to muscle loss. Chronic inflammation, marked by elevated levels of pro-inflammatory cytokines like TNF-*α* and IL-6, is also prevalent in older adults and can exacerbate muscle wasting (Damluji et al. 2023). Gender differences in body composition and fat distribution further influence these metabolic factors. For example, women are more likely to accumulate subcutaneous fat, while men tend to accumulate visceral fat, which is more metabolically active and associated with greater inflammation and IR (Geer and Shen 2009). The interplay between metabolic dysregulation and sarcopenia is complex and influenced by various factors, including lifestyle, genetics, and underlying health conditions. Understanding these age and gender differences is crucial for developing targeted interventions to prevent and manage sarcopenia. Personalized approaches that consider these differences can help optimize muscle health and metabolic function in older adults (Palmer and Jensen 2022)

## 3. Metabolic factors as biomarkers of sarcopenia

According to the EWGSOP2 guidelines the clinical diagnosis of sarcopenia is based on reduced muscle strength along with decreasing muscle mass or quality. Sarcopenia is linked to the three main differential diagnoses of malnourishment, cachexia, and frailty moreover, skeletal muscle insufficiency or failure is the most appropriate method to characterize the sarcopenic condition (Cruz-Jentoft 2016).

The pathophysiology of sarcopenia is influenced by multiple molecular pathways in which the loss of muscle mass and strength with aging is caused by a combination of extrinsic (chronic inactivity, poor nutrition, and endocrine dysfunction) and intrinsic (generalized inflammation, oxidative stress, calcium dysregulation, apoptosis, hypoxia, and the disintegration of neuromuscular junction) factors (Ogawa et al. 2016). Due to the multifactorial origin of sarcopenia which includes the loss of muscle mass and strength in the aged, many molecular biomarkers must be used due to the complicated pathophysiology, therefore a single biomarker-based evaluation of sarcopenia do not adequately characterize the phenotype. The diagnostic potential of six circulating biomarkers associated with different pathophysiological processes of sarcopenia was examined by Qaiser et al. in 2021. and identified that the individual cohorts of healthy controls, COPD and CHF patients, depicted lower levels of growth factors (irisin) and higher levels of protein turnaround (amino-terminal pro-peptide of type III procollagen, P3NP), neuromuscular junction integrity (c-terminal agrin fragment 22 (CAF22), osteonectin (cell–matrix interaction), cellular metabolism (fatty acid-binding protein 3, FABP3), and systemic inflammation (macrophage migration inhibitory factor, MIF). Furthermore, none of the biomarkers examined was suitable for use alone in the diagnosis of sarcopenia (ref). and combining the biomarker data into a single risk score improved the accuracy of the sarcopenia diagnosis (Qaisar et al. 2021).

Good biomarkers are necessary for ordinary clinical practice and the conduct of clinical studies, which assess the effects of innovative treatment regimens on the condition, to accurately diagnose, monitor, and treat sarcopenia. In this sense, biomarkers might offer a consistent and globally equivalent readout for treatment effectiveness (Scharf and Heineke 2012). D.A. Morrow and J.A. de Lemos proposed benchmarks for biomarkers: first, the biomarker must be quantified in a way that is accurate and repeatable, and the assay must be accessible, affordable, and appropriate for high throughput analysis (Morrow and de Lemos 2007). Secondly, the biomarkers must provide fresh data that cannot be acquired from previous testing or a thorough clinical evaluation. Overall, from a clinical point the biomarker must demonstrate a robust association with the disease and its outcome. Thirdly, and perhaps most critically, the ideal biomarker should assist clinicians in managing patients with sarcopenia by assisting in the selection of appropriate therapy, monitoring the course of the disease, and determining whether therapy is necessary. Additionally, as a step toward customized medicine, the biomarker may reveal a particular etiology of sarcopenia and, consequently, require a particular course of treatment (Scharf and Heineke 2012).

Several variables, including nutritional inadequacies, IR, changes in systemic and/or muscular levels of IGF-1, and inflammatory proteins can disrupt muscle homeostasis during acute or chronic diseases including sarcopenia. These elements affect the signaling via the mTOR and muscle proteostasis (Vainshtein and Sandri 2020; Picca et al. 2021). Most of the known serum producers are not muscle-specific and instead produce inflammation (such as tumor necrosis factor-*α* or interleukin-6). Skeletal muscle is the primary site of myostatin expression which is a secreted protein and controls skeletal muscle mass negatively and is linked to transforming growth factor-*β*. Earlier studies demonstrate that myostatin is also expressed in non-skeletal muscle tissues such as fat, serum, and the heart (Scharf and Heineke 2012). In mice, local overexpression of myostatin and peripheral myostatin overexpression (in the heart, for instance) result in a marked decrease in skeletal muscle mass (Lee 2004). Severe sarcopenia may affect the endocrine release of myokines from skeletal muscle, making myokines reliable biomarkers for tracking endocrine muscle function. Irisin is key myokine that is highly regarded among the possibilities in this regard and was only recently discovered (Scharf and Heineke 2012). Irisin has the potential to stimulate signaling via the IGF1/A-protein kinase (Akt)/mTOR pathway by activating AkT and extracellular signal-regulated kinase (Reza et al. 2017). Potential diabetic sarcopenia biomarkers include pentadecanoic acid, 5′-MTA, ADMA, and glutamine. Also, ABC transporters and the mTOR signaling pathway probably play a major role in the development of diabetic sarcopenia (Tan et al. 2023).

In independent older persons clinical studies recommend the use of blood-circulating lipid peroxidation products as biomarkers for sarcopenia. The lipid peroxidation products reported include t MDA (malondialdehyde) + 4-HNE (4-hydroxynonenal), are increased in the plasma of sarcopenic individuals. A higher risk of death has been linked to various types of lipid peroxidation indicators, such as urinary levels of 8-isoprostaglandins F2alpha, which have been linked to more unfavorable clinical circumstances. Similarly, several additional redox biomarkers and inflammatory parameters were closely linked to severe reliance and disability in the elderly (de Gonzalo-Calvo et al. 2011). Other possible biomarkers with sarcopenia includes men’s hemoglobin levels and women’s levels of HDL, C-reactive protein leukocytes, and lymphocytes (Coto Montes et al. 2017). Clinical studies show that the detection of sarcopenia in older population is an emerging field and recent advances allow us to reliably evaluate the stage of and severity of the disease.

## 4. Therapeutic approaches targeting metabolic disturbances

### 4.1. Dietary interventions

Dietary variables, such as the intake of carnosine and beta-alanine, influence blood carnosine levels (Wu 2020). Consequently, the observed negative correlation between carnosine and muscle strength in men could be attributed to reverse causation. For example, elderly individuals with diminished muscle function might have taken carnosine supplements (Zhao et al. 2022). Therefore, dietary interventions that reduce inflammation or oxidation, coupled with increased protein consumption to counteract anabolic resistance, may enhance the muscle protein synthesis response to feeding, thereby preventing or mitigating muscle loss. According to early research, antioxidant vitamins and amino acids with anti-inflammatory or antioxidant qualities likely boost the anabolic reaction triggered by protein consumption (Cholewa et al. 2017).

### 4.2. Resistance training and aerobic exercise

Physical activity is considered the most effective treatment for sarcopenia, as there are currently no approved medications specifically for this condition (Larsson et al. 2019). As a primary intervention for sarcopenia, evidence-based clinical practice guidelines frequently and strongly recommend physical activity (Dent et al. 2018). According to a systematic review, resistance training improves body fat mass, hand-grip strength, knee extension strength, gait speed, and performance on the timed up and go test (Chen et al. 2021). However, another assessment found that aerobic exercise was most successful in enhancing muscle strength and physical performance (Negm et al. 2022). A large, randomized trial suggested that a multicomponent intervention (comprising exercise and nutritional counseling) could reduce the prevalence of mobility impairment among older adults with frailty and sarcopenia. These findings indicate that multicomponent therapies may not fully compensate for the gradual loss of muscle and function experienced by older individuals. Early targeted therapies, including resistance exercise alone or in combination with aerobic exercise or balance training may be required to mitigate the loss of muscle and function that older persons experience during aging (Shen et al. 2023). A recent study found that resistance and aerobic exercise in mice increased muscle fiber size, strength, and endurance. It also changed genes and pathways linked to potassium transport, synaptic transmission, JAK-STAT signaling, and PI3K-Akt signaling. These pathways may be used by BDNF, JAK2, RhoC, Myh6, Stat5a, Tnnc1, and other genes to mediate the positive effects of exercise on sarcopenia (Li et al. 2024).

### 4.3. Pharmacological interventions

#### 4.3.1. Anabolic agents and hormone replacement therapy

A multitude of pre-clinical studies are currently evaluating a variety of potential therapeutic compounds. Exerkines, including interleukin 6, TNF-*α*, interleukin 15, fibroblast growth factor 21, irisin, apelin, and others are being studied to determine their potentials in enhancing physical performance and preventing the loss of muscle mass and strength. Exerkines are demonstrated to exhibit autocrine, paracrine, and endocrine effects following the examination of muscle contraction outcomes, particularly during and post-exercise (Rolland et al. 2023). Research on growth-promoting substances as sarcopenia treatments is gaining significance. It has been confirmed that myostatin inhibitors, testosterone, and selective androgen receptor modulators can augment lean muscle mass. However, further research is necessary to ascertain whether this leads to stronger muscles and improved physical performance in older adults with sarcopenia. Testosterone supplements are the most frequently used drugs for enhancing muscle mass and promoting muscle-protein anabolism (Bahat and Ozkok 2024). BCAAs show promise in the treatment of sarcopenia due to their ability to stimulate protein synthesis and reduce protein degradation. Oral supplements of leucine, isoleucine, and valine (BCAAs) are known to enhance muscle health by activating mTOR, inhibiting Atrogin-1 and MuRF-1 proteins, and preserving protein levels (Mantuano et al. 2023).

#### 4.3.2. Potential benefits and risks

It is important to note that, the outcomes of studies on testosterone replacement therapy in men vary depending on factors such as age, pre-treatment testosterone levels, formulation, and delivery systems of the medication. These diverse variables further complicate the assessment of how the therapy influences physical performance and impairment. Although somewhat therapeutic benefits have been observed, potential side effects, including peripheral edema, gynecomastia, polycythemia, and sleep apnea have also been reported and must not be neglected when considering the pharmacologic option. The possibility that elevated testosterone levels may increase the risk of prostate cancer calls for meticulous planning and monitoring throughout the course of testosterone replacement therapy, marking another significant disadvantage of the hormone therapy (Veronese et al. 2021).

## 5. Limitations and perspectives

Determining the causal relationships between metabolic alterations and sarcopenia presents a challenge due to the multifaceted nature of the condition and the existence of confounding variables. Factors such as hospitalization, comorbidities, and a sedentary lifestyle can singly or altogether accelerate the progression of sarcopenia (Liu and Zhu 2023). There are few long-term studies examining metabolic alterations and their impact on the progression of sarcopenia, which limits our comprehension of the disease’s trajectory and potential therapeutic targets (Gustafsson and Ulfhake 2021). Areas that require further exploration include, but are not limited to, the mechanistic details of the myokines released during and after exercise, as well as insights into their effects both in the presence and absence of mechanical stress on the bones (Gries et al. 2022). To elucidate tryptophan’s function in the progression of sarcopenia, a long-term investigation is required (Dai et al. 2021). Considering the substantial overlap in the disrupted molecular pathways in both sarcopenia and muscular IR, it is necessary to determine whether these conditions are in fact unavoidable comorbidities (Cleasby et al. 2016).

## 6. Conclusion

**Fig. 1.**
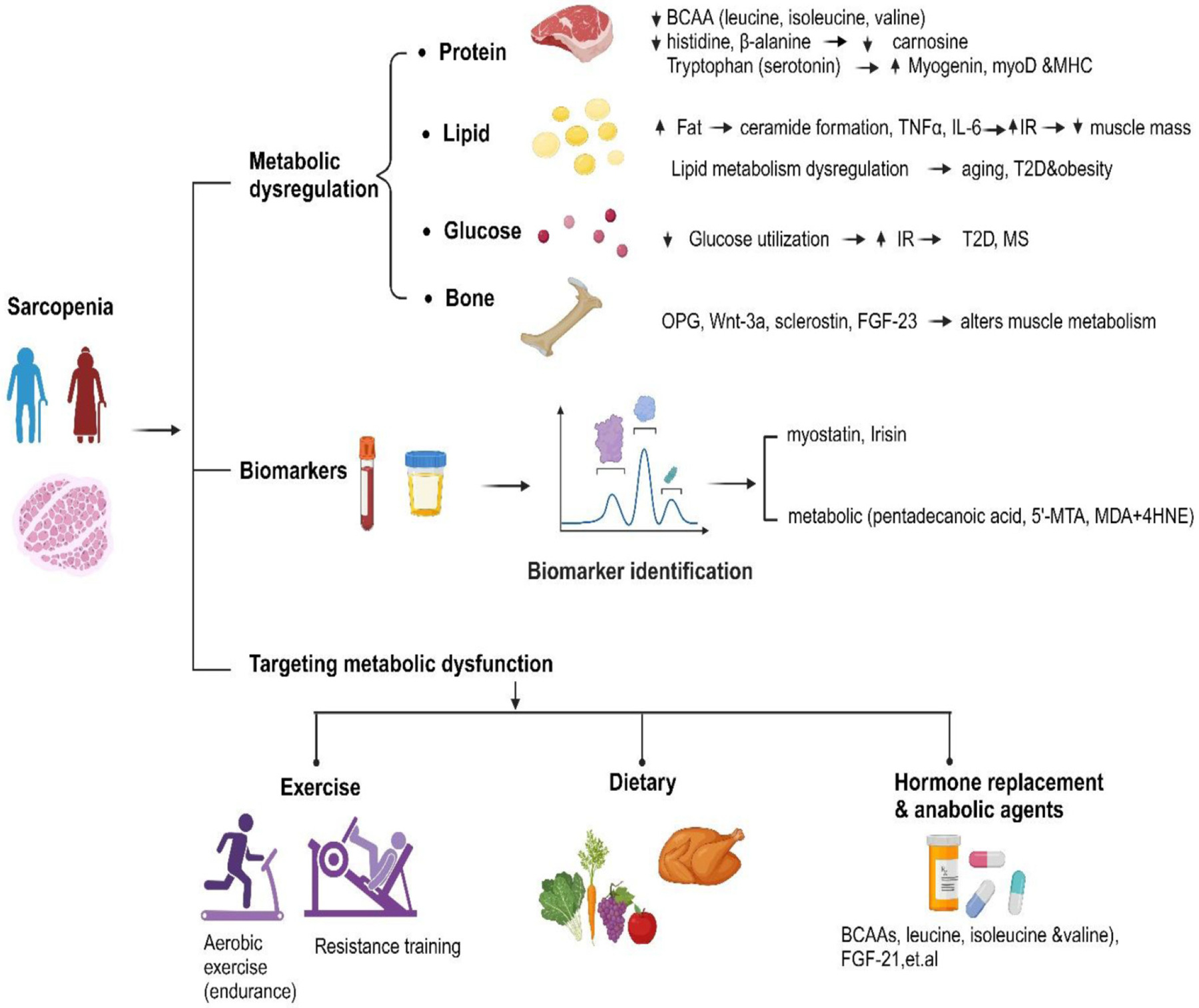
Sarcopenia and associated metabolic dysfunction. The metabolic disturbances were studied at different levels in sarcopenia protein metabolism; decreased blood levels of (BCAAs), particularly leucine, are linked to reduced muscle strength. Tryptophan through its metabolite serotonin can stimulate the expression of myogenic factors and hence the muscle mass increase in muscle fat leads to increase in the ceramide formation and increases the pro-inflammatory cytokines (TNF-*α*, IL-6) that may cause insulin resistance and decrease muscle mass. Fat metabolic dysregulation is also related to aging, T2D and obesity. Disturbances in glucose metabolism leads to decrease glucose utilization which leads to insulin resistance, that could in turn causes T2D and MS bone metabolism: the osteokines including, OPG, wnt-3a, sclerostin, FGF-23 affects muscle metabolism. The metabolites found as sarcopenic markers are pentadecanoic acid, 5′-MTA, MDA + 4HNE). In addition, the blood levels of myostatin and irisin can also indicate sarcopenic susceptibility. Dietary and lifestyle modifications can help counteract sarcopenia, with resistance exercise combined with leucine showing promise in stimulating myogenesis. BCAA, branched-chain amino acids; TNF-*α*, tumor necrosis factor alpha; IL-6, interleukin-6; T2D, type 2 diabetes; IR, insulin resistance; MS, metabolic syndrome; OPG, osteoprotegerin; FGF-23, fibroblast growth factor-23; 5′-MTA, 5′-methylthioadenosine; MDA, malondialdehyde; 4HNE, 4-hydroxynonenal.

In conclusion, earlier studies reveal a robust association between metabolic disorders such as obesity, IR, and sarcopenia. Mitochondrial dysfunction and oxidative stress are two key factors in the pathogenesis of sarcopenia. Research underscores the importance of metabolic factors in the onset of sarcopenia, with potential implications for both intervention strategies and diagnostic procedures. To comprehend the pathogenesis of sarcopenia, it is important to consider the following specific alterations in metabolic changes (Fig. 1). (i) The amino acid profiles, including aspartic acid, glutamic acid, and leucine, that are associated with changes in muscle mass and strength. Furthermore, metabolites such as citrulline and tryptophan have been linked to the severity of sarcopenia. (ii) The lipid profile. Although sarcopenia is not always associated with weight loss, an increase in fat mass can coincide with muscle deterioration. Given that skeletal muscle relies on FAs for contraction, it is essential for the metabolism of energy. Inflammation and dyslipidemia can potentially exacerbate sarcopenia. Maintaining normal parameters of lipid metabolism could help prevent sarcopenia. Certain lipid subfractions such as, ceramides, DAGs, and triglycerides have been associated with the development of sarcopenia, suggesting potential preventative measures. Inflammation is a consequence of altered lipid signaling pathways in sarcopenic muscle. Inflammation and IR may also contribute to the accumulation and dysfunction of lipids in muscle tissue. Understanding these pathways is crucial for targeted therapies aimed at sarcopenia. (iii) Glucose metabolism is largely dependent on skeletal muscle, and both sarcopenia and aging exacerbate IR. Particularly in men, metabolic disorders such as diabetes and MS are associated with muscle loss. Factors like exercise, gut microbiome, and dietary choices significantly influence muscle function and glucose regulation. Molecular mediators that impact overall metabolic health include insulin, IGF1, amino acids, and myostatin. These mediators regulate glucose and protein metabolism in skeletal muscle. (iv) Bone metabolism is closely linked with skeletal muscle; therefore, osteopenia/osteoporosis and sarcopenia often coexist in older adults, heightening the risk of fractures and falls. Diagnostic techniques including CT scan offer promising avenues for identifying and treating both disorders. Promising pathways for diagnosis have been shown by recent studies investigating molecular biomarkers. Accuracy is improved when biomarker data are combined. Myokines and myostatin show up as possible targets, providing information on how muscles work. Promising early markers include inflammatory parameters and lipid peroxidation products. In addition, regarding therapeutic intervention, resistance training, and creatine supplementation may help reduce bone catabolism markers while increasing lean mass and muscle strength. Supplementation and exercise training may enhance protein synthesis and mitigate muscle atrophy. Exercise is crucial for augmenting bone mass and strength as it influences the release of myokines from the muscles, such as irisin and BAIBA, which rgulate bone metabolism. A deeper understanding of the skeletal muscle secretome and myokine-mediated communication between muscle and bone paves the way for innovative therapeutic strategies for metabolic diseases.

## Data Availability

This manuscript does not report data.
